# Associations of body roundness index and body mass index with obstructive sleep apnea: evidence from two cohorts study

**DOI:** 10.3389/fneur.2025.1709205

**Published:** 2025-12-16

**Authors:** Lan Lan, Yuenan Ni, Yubei Zhou, Fengming Luo

**Affiliations:** 1 Department of Respiratory and Critical Care Medicine, West China Hospital, Sichuan University, Chengdu, Sichuan, China; 2 State Key Laboratory of Respiratory Health and Multimorbidity, West China Hospital, Sichuan University, Chengdu, Sichuan, China

**Keywords:** obstructive sleep apnea, obesity, body roundness index, body mass index, visceral adiposity

## Abstract

**Background:**

Obstructive sleep apnea (OSA) is closely associated with obesity. Traditional anthropometric indices body mass index (BMI) could not assess body fat content precisely. The body roundness index (BRI) has emerged as a novel adiposity metric reflecting central fat distribution. This study aimed to compare BRI and BMI in assessing the risk and worsening of OSA severity in community-based cohort study.

**Methods:**

We analyzed data from two well-characterized cohorts: the Osteoporotic Fractures in Men Study (MrOS) and the Sleep Heart Health Study (SHHS). Statistical and machine learning approaches were used to assess associations between BRI, BMI, and OSA. Sex-stratified analyses and models evaluating of future OSA severity categories using baseline features were also performed.

**Results:**

BRI showed stronger associations with severe baseline OSA than BMI in both cohorts (MrOS: OR = 1.50 vs. 1.22, SHHS: OR = 1.21 vs. 1.12). BRI demonstrated significant dose–response trends across quartiles, while BMI was only significant in the highest quartile. RCS analyses indicated linear relationships between both BRI, BMI, with severe baseline OSA. A significant sex interaction was observed (P for interaction = 0.015), with a stronger association in men (OR = 1.44, 95% CI: 1.16–1.66) than in women (OR = 1.13, 95% CI: 1.02–1.26). Random forest models using baseline features showed higher AUCs for BRI than BMI in assessing worsening of follow-up OSA severity (MrOS: 0.67 vs. 0.57, *p* < 0.05, SHHS: 0.73 vs. 0.68, *p* < 0.01). SHAP interpretation confirmed BRI as a superior feature, with greater feature contribution than BMI.

**Conclusion:**

BRI showed closer associations with severe baseline OSA than BMI and worsening OSA severity during follow-up in two community-based cohorts. These findings suggest that BRI may serve as a useful anthropometric marker for identifying individuals at higher risk of severe or worsening OSA.

## Instruction

Obstructive sleep apnea (OSA) is a common and increasingly recognized sleep disorder, particularly prevalent among middle-aged and older adults. Globally, it is estimated that approximately 936 million adults aged 30–69 are affected by OSA, with 425 million experiencing moderate to severe forms of the disease (1). OSA is characterized by recurrent episodes of partial (hypopnea) or complete (apnea) upper airway obstruction during sleep, leading to intermittent hypoxemia, sympathetic nervous system activation, and sleep fragmentation. These pathophysiological disturbances have been strongly associated with increased risks of cardiovascular disease, type 2 diabetes mellitus, stroke, premature mortality, and diminished quality of life (2).

Among the various risk factors identified, obesity is one of the most significant and well-established contributors to the development and progression of OSA (3). Clinical observations consistently reveal a high prevalence of obesity among individuals diagnosed with OSA in outpatient and population-based cohorts (4). Foster et al. (5, 6) reported that 87% of obese patients with diabetes had clinically significant OSA, indicating the significant contribution of obesity to disease risk. Furthermore, epidemiological studies have consistently shown a graded increase in the prevalence of OSA as BMI, or other measures of body habitus, increases in adults. It is estimated that approximately 60–90% of adults with OSA are overweight or obese (7). Importantly, weight reduction has been shown to markedly alleviate OSA severity (8). Recent meta-analysis assessing the effect of surgical weight loss on measures of OSA in whom the pooled AHI was reduced by 38.2 events/h to a final value of 15.8 events/h, highlighting the therapeutic potential of weight management in OSA treatment (9). In addition to bariatric surgery, pharmacologic agents targeting obesity have emerged as promising therapeutic options for OSA. Glucagon-like peptide-1 receptor agonists and dual GIP/GLP-1 receptor agonists have been shown to induce substantial weight loss and meaningful reductions in AHI in recent randomized trials (10). Malhotra et al. (11) reported that tirzepatide led to significant improvements in sleep-disordered breathing, hypoxic burden, and cardiometabolic profiles in adults with obesity and moderate-to-severe OSA. These emerging therapeutic options further highlight the importance of assessing adiposity and fat distribution when evaluating OSA risk.

Obesity predisposes to OSA and the prevalence of OSA is increasing worldwide, because of the ongoing epidemic of obesity (12, 13). However, current approaches to assessing obesity, particularly using body mass index (BMI), are limited in distinguishing fat distribution and accurately capturing visceral adiposity, which is essential in the pathogenesis of OSA. Given these limitations, it is essential to identify the most accurate obesity indices for effective diagnosis and management of OSA.

In recent years, novel anthropometric measures such as a body shape index (ABSI), weight-adjusted waist index (WWI) and body roundness index (BRI) have emerged. Among them, BRI, a simple index derived from height and waist circumference, has shown superior performance in estimating visceral fat accumulation and predicting adverse outcomes, including cardiovascular disease and all-cause mortality (14, 15). Given the established role of visceral fat in the pathophysiology of OSA (16), BRI may serve as a more sensitive and physiologically relevant marker for identifying individuals at increased risk. However, the potential utility of BRI in OSA risk assessment remains insufficiently explored.

In this study, we investigated the association between BRI and both the presence and follow-up worsening severity of OSA, using data from two large, well-characterized community-based cohorts. The performance of BRI and BMI was directly compared in both cross-sectional and follow-up analyses assessing follow-up OSA severity based on baseline features. Our findings may provide new insights into the clinical utility of BRI for improving OSA risk stratification and informing early intervention strategies.

## Method

### Study design and population

The Osteoporotic Fractures in Men Study (MrOS) is a community based cohort initiated in 2000, designed to investigate risk factors for fractures and other aging-related outcomes in older men. It enrolled 2,911 community-dwelling men aged 65 years or older across multiple clinical centers in the United States. Detailed inclusion and exclusion criteria for MrOS have been described in previously published studies. A MrOS Sleep Study follow-up visit was performed in 1,026 participants on average 6.0 ± 1.0 years after the baseline MrOS sleep visit (2003–2005), with follow-up assessments conducted between 2009 and 2012 (17, 18).

The Sleep Heart Health Study (SHHS) is a multicenter cohort sponsored by the National Heart, Lung, and Blood Institute (NHLBI), enrolling 6,441 adults aged ≥40 years between 1995 and 1998 to evaluate cardiovascular consequences of sleep-disordered breathing. A follow-up PSG was performed 5.0 ± 0.5 years later (range 4.5–5.5 years) in 3295 subjects during SHHS Visit 2 conducted between 2001 and 2003 (19, 20).

**Figure 1:**
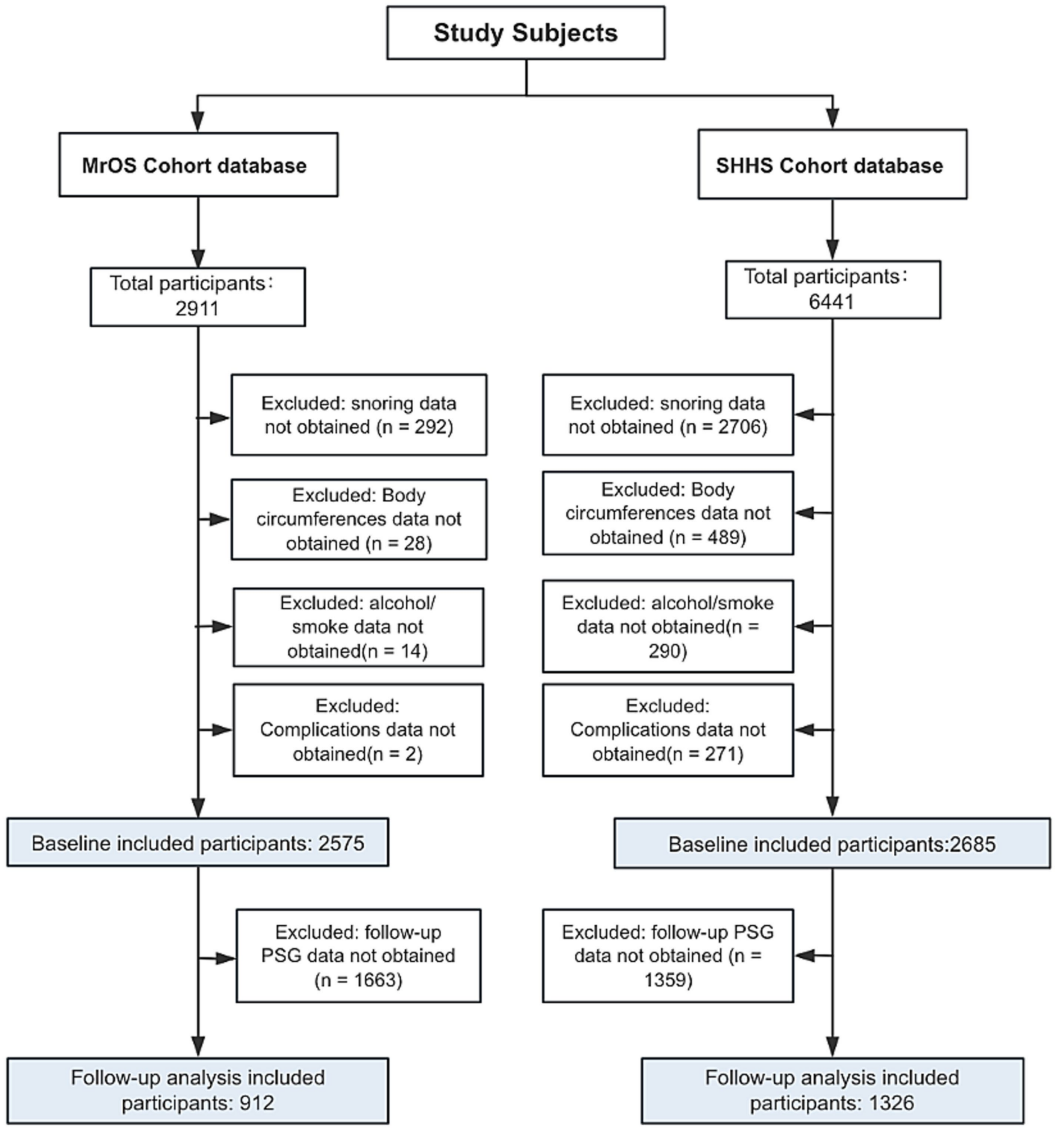
Flowchart of participants selection. MrOS, Osteoporotic Fractures in Men Study; SHHS, Sleep Heart Health Study.

For the present analysis, we included participants with complete data on OSA status, height, waist circumference, and other key anthropometric and covariate variables. Participants with missing or incomplete data were excluded. The final analytic sample size, along with the detailed inclusion and exclusion criteria for both baseline and follow-up assessments, is presented in Figure 1 and Supplementary Figure 1.

### Calculation of anthropometric indices

The body roundness index (BRI) was calculated using the following formula:


$$\mathrm{BRI}=364.2-365.5\times {\left\{1-{\left(\mathrm{WC}\;\left(\mathrm{cm}\right)/\left(2\pi \right)/\left(0.5\times \mathrm{height}\;\left(\mathrm{cm}\right)\right)\right)}^{2}\right\}}^{0.5}$$

A body mass index (BMI), was calculated using the formula:


$$\mathrm{BMI}=\mathrm{Weight}\;\left(\mathrm{kg}\right)/{\left(\mathrm{height}\;\left(m\right)\right)}^{2}$$

### Diagnosis of probable OSA and worsening severity of OSA

OSA events were assessed using overnight polysomnography. OSA is defined by the number of obstructive apnea and hypopnea episodes per hour of sleep (apnea-hypopnea index, AHI), reflecting the degree of departure from the normal physiology of breathing during sleep. According to the AASM criteria, apnea was defined as a complete cessation of airflow for ≥10 s, and hypopnea as a ≥ 30% reduction in airflow lasting ≥10 s, accompanied by either a ≥ 3% oxygen desaturation or an arousal (21). The severity of OSA was determined based on the AHI: an AHI of 5–15 events per hour was classified as mild OSA, 15–30 as moderate OSA, and ≥30 as severe OSA (22). OSA *severity worsening* was defined as an increase in severity category at follow-up compared with baseline according to AHI categories based on standard thresholds (delta OSA severity grade > 0). The change in severity was calculated as:


$$\begin{array}{l} \Delta \mathrm{OSA severity grade}=\mathrm{OSA}\;{\mathrm{severity grade}}_{\mathrm{follow}-\mathrm{up}}\\ \;\;\;\;\;\;\;\;\;\;\;\;\;\;\;\;\;\;\;\;\;\;\;\;\;\;\;\;\;\;\;\;\;\;\;\;\;\;\;\;\;\;\;\;\;\;\;\;\;\;-\mathrm{OSA}\;{\mathrm{severity grade}}_{\mathrm{baseline}} \end{array}$$

OSA *severity worsening* was defined as:


ΔOSA severity grade>0

### Covariates

To adjust for potential confounding factors, several covariates were included in the multivariable-adjusted models. These covariates included demographic variables (age, gender, race), lifestyle factors (smoking status and alcohol consumption), and health conditions (hypertension) (Table 1).

**Table 1:** Basic characteristics of the population in MrOS study.

Characteristic variable	Non-osa	Mild-osa	Moderate-osa	Severe-osa	*p*
Subjects, *n*	232	906	766	635	
Age, y	75.70 (5.47)	75.96 (5.32)	75.82 (5.28)	77.08 (5.35)	<0.001
Hypertension	119	459	431	395	0.006
Snore	180	781	678	611	<0.001
Smoke	7	18	18	7	0.177
Drink	153	610	504	436	0.796
Tired	184	714	625	551	0.313
Race					
White	210	825	709	615	0.658
African American	10	24	23	18	
Asian	8	34	16	20	
Other races	4	23	18	18	
Weight	79.75 (11.89)	80.76 (12.52)	82.44 (12.38)	85.40 (13.56)	<0.001
Ess score	5.90 (3.73)	5.93 (3.57)	6.26 (3.66)	6.47 (3.86)	0.11
BMI (Kg/m^2^)	25.95 (3.17)	26.57 (3.60)	27.31 (3.56)	28.42 (3.94)	<0.001
BRI	4.47 (1.24)	4.69 (1.30)	4.94 (1.29)	5.39 (1.40)	<0.001
Neck circumference (cm)	38.76 (2.64)	39.02 (2.70)	39.56 (2.73)	40.29 (2.85)	<0.001
Waist circumference (cm)	96.68 (10.42)	98.09 (10.58)	99.69 (10.02)	103.01 (10.66)	<0.001
Hip circumference (cm)	101.17 (7.58)	102.06 (8.20)	102.95 (7.85)	104.76 (8.53)	<0.001
Total sleep time (min)	355.95 (67.10)	361.28 (67.40)	356.50 (67.47)	346.14 (74.23)	<0.001
Sleep time with SaO_2_ < 90%	0.95 (4.60)	2.03 (7.13)	4.08 (8.76)	8.52 (12.09)	<0.001
N1	5.74 (3.37)	6.10 (3.45)	6.84 (4.05)	8.51 (5.63)	<0.001
N2	61.34 (10.69)	61.77 (9.28)	62.06 (9.61)	65.78 (9.22)	<0.001
N3	12.67 (9.61)	12.05 (8.99)	11.42 (9.04)	9.16 (8.24)	<0.001
REM sleep stage (% of total sleep time)	20.32 (6.71)	20.21 (6.38)	19.84 (6.48)	16.67 (6.58)	<0.001
Sleep efficacy	78.42 (11.58)	78.00 (11.59)	76.07 (11.60)	72.47 (12.71)	<0.001
Min SaO_2_, %	89.42 (3.08)	86.60 (4.28)	83.39 (6.31)	80.13 (7.19)	<0.001
Height (cm)	174.99 (6.86)	174.19 (6.73)	173.55 (6.62)	173.16 (6.54)	0.001
Arousal index	15.53 (7.69)	18.52 (8.04)	23.85 (8.81)	33.94 (13.28)	<0.001
Diabetes	20	105	99	109	0.008
Heart failure	6	41	43	61	<0.001

MrOS, Osteoporotic Fractures in Men Study; OSA, obstructive sleep apnea; BMI, body mass index; BRI, body roundness index; SaO_2_, arterial oxygen saturation; REM, rapid eye movement.

### Statistical analysis

#### Descriptive analysis

Continuous variables were described as mean ± SD or median (IQR) depending on normality and compared using independent *t*-tests or Wilcoxon rank-sum tests as appropriate. Normality was assessed using the Shapiro–Wilk test and visual inspection of histograms. Categorical variables were summarized as counts and percentages, with group comparisons conducted using the chi-square test or Fisher’s exact test. For comparisons across multiple OSA severity groups, continuous variables were analyzed using one-way ANOVA or the Kruskal–Wallis test, while categorical variables were compared using the chi-square test. When multiple comparisons were performed, *p*-values were adjusted using Bonferroni correction. All statistical analyses were conducted using R software, version 4.3.0. Two-sided *p*-values less than 0.05 indicate statistical significance.

#### Baseline cross-sectional analyses

To assess the association between the BRI, BMI, and baseline severe OSA, we constructed three logistic regression models with progressively increasing levels of covariate adjustment: Model 1 was unadjusted. Model 2 was further adjusted for age, race/ethnicity, gender, snore. Model 3 incorporated additional variables, including age, race/ethnicity, gender, neck circumference, snoring, tiredness, smoking status, alcohol use, hypertension, and history of diabetes (23). This consistent stepwise adjustment strategy was designed to control for potential confounders and to assess the robustness and power of the anthropometric indices across different modeling approaches. Subgroup analyses were conducted by stratifying the population based on gender, age, and other key variables.

We applied restricted cubic spline (RCS) curves to investigate the potential nonlinear relationship between BRI or BMI and outcome events. Furthermore, we performed subgroup analyses to verify the reliability of the findings. To control the risk of false positives due to multiple hypothesis testing, the Bonferroni correction method was used in this study.

#### Follow-up analyses

Follow-up analyses evaluated the worsening of OSA severity from baseline to follow-up. ΔOSA severity grade was calculated as the difference in severity category between follow-up and baseline. Baseline features of change in severity were analyzed using regression models and subsequently incorporated into machine learning–based frameworks described below.

### Construction and performance evaluation of machine learning model

Covariates included in Models 1, 2, and 3 were selected based on prior clinical relevance and were further informed by feature importance analyses using LASSO regression and the Boruta algorithm. Feature selection was performed using the SHHS cohort, which has a larger and more comprehensive dataset with both sexes. Selected variables were used to develop and validate models separately in SHHS and MrOS to assess external generalizability. This approach ensured that the choice of adjustment variables was systematic and data-driven rather than arbitrary.

To compare the performance of BRI and BMI for worsening OSA severity during follow-up under different adjustment strategies, patients were randomly divided into training (80%) and test (20%) sets. Random forest (RF) models were developed using the selected covariates and followed the same stepwise adjustment strategy as the logistic regression models (Models 1, 2, and 3). RF hyperparameters are reported in Supplementary Table 1. To address selective loss to follow-up over approximately 5 years, inverse probability weighting (IPW) was applied. Probabilities of (1) survival to follow-up and (2) completion of follow-up PSG were estimated using logistic regression based on baseline sleep measures and covariates. Weights were generated by multiplying the marginal probability of follow-up and were winsorized at the 1st and 99th percentiles to reduce the influence of extreme values. Details of the weighting strategy are provided in the Supplementary material.

The modeling performance of BRI and BMI was evaluated based on the area under the receiver operating characteristic curve (AUROC). To further assess the incremental value of BRI and BMI, category-free net reclassification improvement (NRI) was calculated in both cohorts, following recommended approaches for evaluating improvements in risk stratification, detailed procedures are provided in the Supplementary material. Additionally, the interpretability of the final model was investigated using SHapley Additive exPlanations (SHAP) to interpret variable contributions.

## Results

### Baseline characteristics of study individuals

A total of 2,575 participants who met the inclusion criteria from the MrOS were included in this study. Of these, 232were classified as non-OSA, 906 as mild OSA, 766 as moderate OSA, and 635 as severe OSA (Table 1). 2,685 participants from the SHHS who fulfilled the inclusion criteria were included. Among them, 372 were classified as non-OSA, 972 as mild OSA, 834 as moderate OSA, and 530 as severe OSA (Table 2). The participant selection process for both the MrOS and SHHS databases is illustrated in Figure 1, detailing the sequential exclusion criteria. Ultimately, 2,575 participants from MrOS and 2,685 participants from SHHS were included in the final analysis. Follow-up PSG was obtained in 912 MrOS participants and 1,326 SHHS participants, who were included in the follow-up analyses. The full inclusion and exclusion flowchart for both cohorts are shown in Figure 1.

**Table 2:** Basic characteristics of the population in the Sleep Heart Health Study (SHHS).

Characteristic variable	Non-osa	Mild-osa	Moderate-osa	Severe-osa	*p*
Subjects, *n*	372	972	834	530	
Age, y	59.59 (10.62)	62.16 (9.64)	64.38 (9.92)	65.14 (9.72)	<0.001
Height	165.74 (9.75)	167.41 (9.32)	169.92 (9.22)	170.77 (8.78)	
Hypertension	111	367	385	266	<0.001
Snore	340	954	821	521	0.69
Smoke	185	502	469	310	0.048
Drink	176	477	394	248	0.633
Gender, male	110	437	558	395	<0.001
Tired	326	900	767	495	0.73
Race					
White	286	868	742	478	<0.001
African American	31	49	47	34	
Asian	32	55	45	18	
Weight	71.28 (13.85)	77.11 (14.53)	82.87 (14.94)	88.70 (16.05)	<0.001
Ess score	7.09 (4.20)	7.51 (4.24)	7.98 (4.36)	8.71 (4.81)	<0.001
BMI (kg/m^2^)	26.04 (4.30)	27.88 (4.68)	28.96 (4.81)	30.92 (5.32)	<0.001
BRI	4.43 (1.65)	4.97 (1.74)	5.38 (1.70)	5.89 (1.81)	<0.001
Neck circumference (cm)	35.59 (3.55)	37.47 (3.86)	39.34 (3.77)	40.84 (3.95)	<0.001
Waist circumference (cm)	90.79 (12.47)	95.98 (12.44)	100.58 (11.83)	104.92 (12.42)	<0.001
Hip circumference (cm)	102.74 (9.48)	105.07 (9.93)	107.42 (36.27)	108.92 (12.18)	<0.001
Total sleep time (min)	374.79 (58.41)	368.992 (60.10)	356.11 (61.11)	348.27 (66.07)	<0.001
Sleep time with SaO_2_ < 90%	0.40 (3.08)	1.75 (7.67)	3.51 (9.93)	10.18 (15.50)	<0.001
N1	4.70 (3.38)	5.08 (3.43)	5.84 (3.85)	6.93 (5.05)	<0.001
N2	54.24 (11.34)	55.33 (11.07)	57.51 (11.30)	61.42 (11.35)	<0.001
N3	20.21 (11.49)	18.97 (11.65)	16.83 (11.52)	13.70 (10.84)	<0.001
REM sleep stage (% of total sleep time)	20.84 (6.26)	20.61 (6.05)	19.83 (5.88)	17.95 (6.34)	<0.001
Sleep efficacy	85.55 (8.55)	84.35 (9.45)	82.00 (10.24)	80.17 (11.63)	<0.001
Min SaO_2_, %	90.05 (3.09)	87.35 (4.34)	84.24 (4.85)	79.27 (7.88)	<0.001
Height (cm)	165.74 (9.75)	167.41 (9.32)	169.92 (9.22)	170.77 (8.78)	<0.001
Arousal index	12.59 (5.86)	16.03 (6.63)	20.38 (7.99)	30.74 (14.55)	<0.001
Diabetes	10	53	72	52	<0.001
Heart failure	3	11	17	11	0.22

SHHS, Sleep Heart Health Study; OSA, obstructive sleep apnea; BMI, body mass index; BRI, body roundness index; SaO_2_, arterial oxygen saturation; REM, rapid eye movement.

### Association between BRI, BMI, and severe baseline OSA

To investigate the association between severe baseline OSA and BRI, BMI, three multivariable logistic regression models (Table 3) and random forest models (Supplementary Tables 1, 2) were constructed in the MrOS and SHHS cohorts. In the Model 1, multiple logistic regression analysis suggested that there existed a significant positive correlation between baseline OSA and BRI. After the adjustment in Model 3, BRI and BMI were significantly associated with severe baseline OSA (BRI: OR = 1.36, 95% CI 1.23–1.50, *p* < 0.001; BMI: OR = 1.12, 95% CI 1.08–1.17, *p* < 0.001) in the MrOS cohort. BRI showed significant associations with baseline OSA across all quartiles, while BMI, only the highest quartile (Q4) showed a significant association with baseline OSA. In the SHHS cohort, similar associations were observed, with BRI (OR = 1.21, 95% CI: 1.15–1.27, *p* < 0.001), showing a stronger association than BMI (OR = 1.12, 95% CI: 1.10–1.15, *p* < 0.001). In sensitivity analyses using continuous AHI, BRI showed stronger positive associations with AHI than BMI in both MrOS and SHHS (Supplementary Table 11).

**Table 3:** Associations of body roundness index with severe baseline OSA in MrOS cohort and SHHS cohort.

MrOS cohort	OR (95% CI), *p*-value					
	BRI			BMI		
	Model 1	Model 2	Model 3	Model 1	Model 2	Model 3
Continuous	1.41 (1.25,1.58)***	1.30 (1.16,1.47)***	1.36 (1.23,1.50)***	1.13 (1.08,1.18)***	1.76 (1.35,2.31)***	1.12 (1.08,1.17)***
Categories						
Q1	Reference	Reference	Reference	Reference	Reference	Reference
Q2	1.09 (1.02,1.17)*	1.09 (1.02,1.18)*	1.06 (1.02,1.12)*	1.06 (0.99,1.13)	1.06 (0.99,1.14)	1.06 (0.98, 1.14)
Q3	1.14 (1.07,1.23)***	1.14 (1.05,1.23)**	1.07 (1.01,1.12)**	1.07 (0.99,1.15)	1.08 (0.99,1.17)	1.06 (0.97,1.16)
Q4	1.21 (1.13,1.30)***	1.21 (1.09,1.33)**	1.16 (1.09, 1.23)***	1.20 (1.12, 1.28)***	1.22 (1.09,1.36)***	1.14 (1.08, 1.22)***
P for trend	<0.001	<0.001	<0.001	<0.001	0.001	0.004

SHHS cohort	OR (95% CI), *p*-value					
	BRI			BMI		
	Model 1	Model 2	Model 3	Model 1	Model 2	Model 3
Continuous	1.23 (1.14,1.33)***	1.23 (1.14,1.34)***	1.21 (1.15,1.27)***	1.13 (1.09,1.17)***	1.12 (1.08,1.17)***	1.12 (1.10,1.15)***
Male	1.49 (1.35,1.65)***	1.52 (1.37, 1.68)***	1.44 (1.16,1.66)***	1.15 (1.13,1.18)***	1.16 (1.13,1.19)***	1.15 (1.10,1.20)***
Female	1.34 (1.23,1.46)***	1.34 (1.22,1.47)***	1.13 (1.02,1.26)***	1.08 (1.09,1.11)***	1.14 (1.12,1.17)***	1.10 (1.05,1.15)***
Categories						
Q1	Reference	Reference	Reference	Reference	Reference	Reference
Q2	1.02 (0.98, 1.06)	1.01 (0.98, 1.05)	1.01 (0.98, 1.05)	1.00 (0.96,1.03)	0.99 (0.95, 1.03)	0.99 (0.959,1.03)
Q3	1.02 (0.98, 1.06)	1.01 (0.97, 1.04)	1.01 (0.97, 1.05)	1.03 (0.99,1.07)	1.01 (0.98,1.05)	1.01 (0.98,1.05)
Q4	1.12 (1.07,1.17)**	1.11 (1.06,1.16)**	1.15 (1.10,1.21)***	1.13 (1.08, 1.19)**	1.12 (1.07,1.17)**	1.14 (1.08,1.20)***
P for trend	<0.001	<0.001	<0.001	<0.001	<0.001	<0.001

Model 1 was unadjusted. Model 2 was adjusted for age, race, snore. Model 3 was fully adjusted for age, race/ethnicity, gender, neck circumference, snoring, tiredness, smoking status, alcohol use, hypertension, and history of diabetes. *p < 0.05, **p < 0.001, ***p < 0.0001.

Continuous variables were modeled per 1-unit increase in BRI or BMI. Quartile categories (Q1–Q4) were defined using cohort-specific distributions, with Q1 as the reference group. OSA, obstructive sleep apnea; MrOS, Osteoporotic Fractures in Men Study; BRI, body roundness index; BMI, body mass index; OR, odds ratio; CI, confidence interval.

### Detection of linear relationships

**Figure 2:**
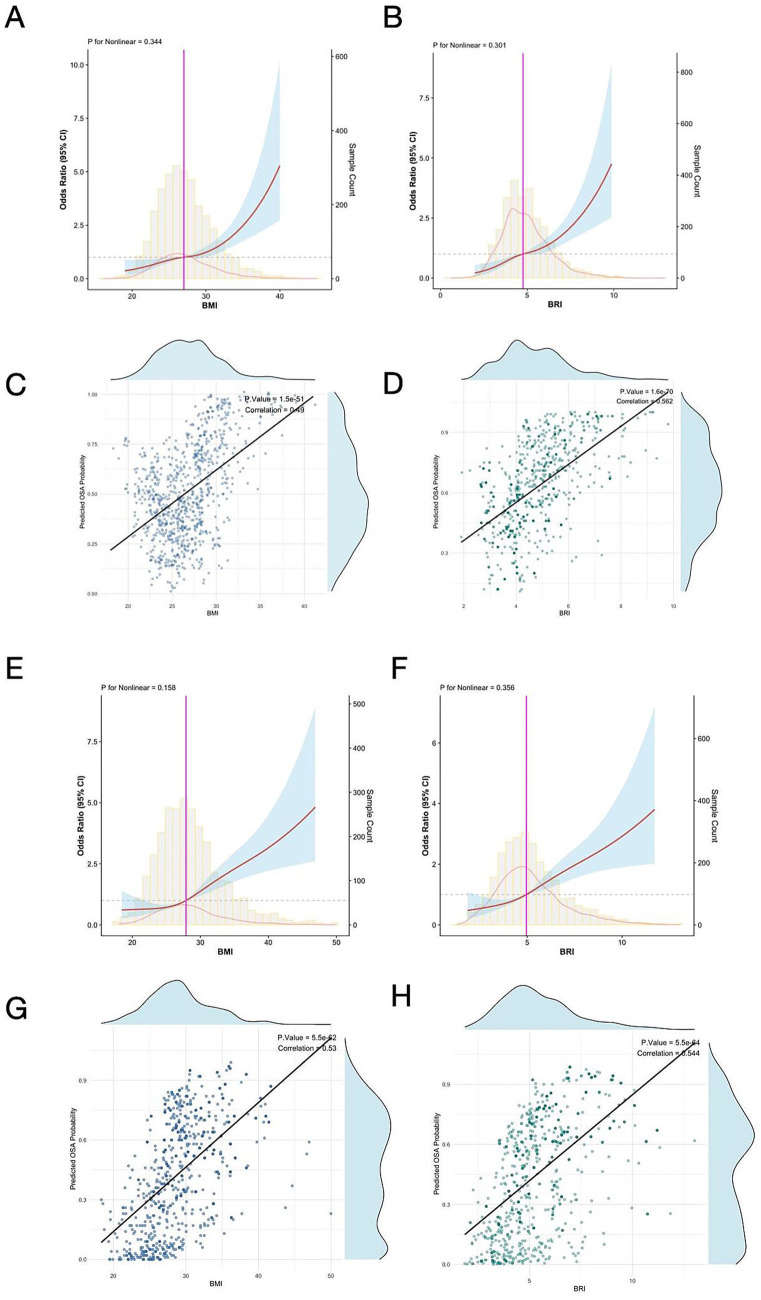
Restricted cubic spline analysis illustrating the association between BMI **(A)**, BRI **(B)**, and severe baseline OSA risk in the MrOS cohort. Linear correlation scatterplots for estimated baseline OSA probability with BMI **(C)** and BRI **(D)**. Restricted cubic spline analysis illustrating the association between BMI **(E)**, BRI **(F)**, and severe baseline OSA risk in the SHHS cohort. Linear correlation scatterplots for estimated OSA probability with BMI **(G)** and BRI **(H)**. RCS, restricted cubic spline; BMI, body mass index; BRI, body roundness index; OSA, obstructive sleep apnea; MrOS, Osteoporotic Fractures in Men Study; SHHS, Sleep Heart Health Study.

RCS analysis demonstrated linear relationships (P for nonlinearity > 0.05) between BMI, BRI, and severe baseline OSA risk in both the MrOS and SHHS cohorts (Figures 2A,B,E,F). In the MrOS cohort, linear correlation scatterplots showed that BMI and BRI were positively correlated with the estimated probability of severe baseline OSA (BMI: *p* < 0.001, Correlation = 0.45; BRI: *p* < 0.001, Correlation = 0.562) (Figures 2C,D). Similarly, in the SHHS cohort, both BMI and BRI exhibited significant positive correlations with the estimated probability of severe baseline OSA (BMI: *p* < 0.001, Correlation = 0.53; BRI: *p* < 0.001, Correlation = 0.544) (Figures 2G,H).

### Subgroup analyses

**Figure 3:**
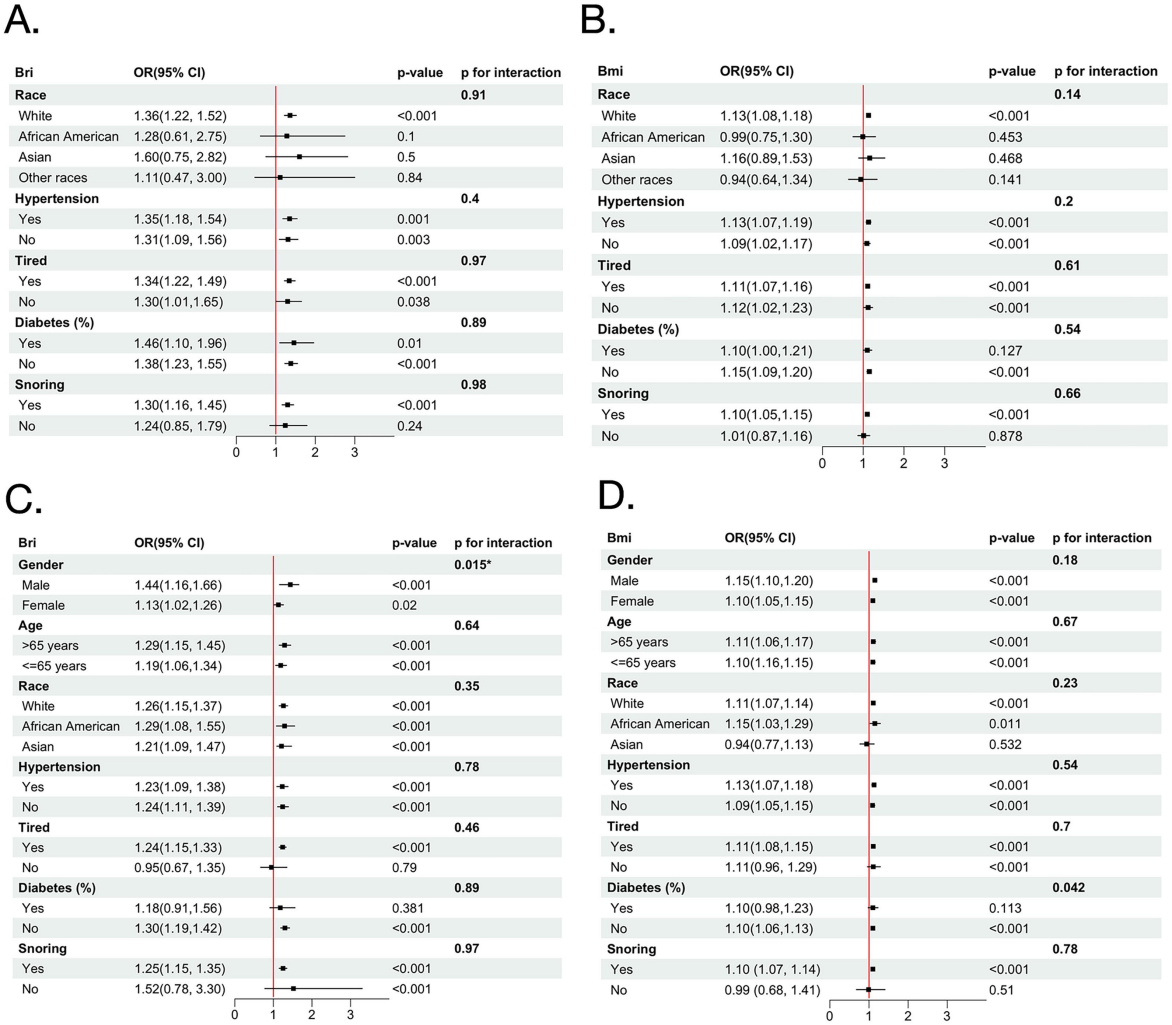
Forest plots illustrating stratified analyses of the association between BRI and severe baseline OSA **(A)**, and BMI and severe baseline OSA **(B)** in the MrOS cohort; and BRI **(C)** and BMI **(D)** in the SHHS cohort. BMI, body mass index; BRI, body roundness index; OSA, obstructive sleep apnea; MrOS, Osteoporotic Fractures in Men Study; SHHS, Sleep Heart Health Study; OR, odds ratio.

Multiple subgroup analyses and interaction tests were conducted to assess the associations of BRI and BMI with baseline OSA (Figure 3). BRI remained significantly associated with baseline OSA across gender and age subgroups. A significant interaction was observed for gender (P for interaction = 0.015), with a stronger association in males (OR = 1.44, 95% CI: 1.16–1.66) than in females (OR = 1.13, 95% CI: 1.02–1.26). BMI showed significant associations in all subgroups without significant interactions for gender or age. Stratified analyses examined whether the associations of BRI and BMI differed across cohorts, and no significant interactions were observed, indicating no evidence of between-cohort heterogeneity (Supplementary Table 1).

**Figure 4:**
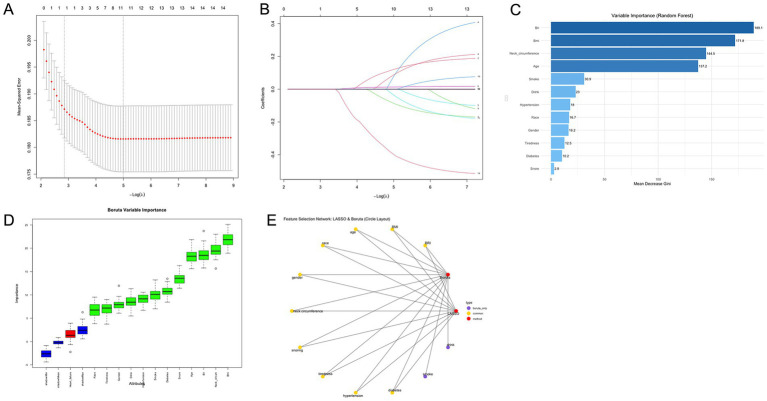
OSA feature selection chart. **(A)** Path diagram and coefficient trajectories from LASSO regression analysis. **(B)** Cross-validation plot for LASSO regression showing the optimal lambda value and corresponding model error. **(C)** Variable importance ranking from random forest analysis including BRI and BMI. Cross-validation plot for LASSO regression showing the optimal lambda value and corresponding model error. **(D)** Feature importance ranking based on the Boruta algorithm. The horizontal axis shows the variable names, and the vertical axis represents the importance score. The box plots display the distribution of importance scores during model iterations. **(E)** Feature selection network diagram. The yellow section shows the results of the LASSO regression analysis, the red section shows the results of the Boruta algorithm, and the purple section shows the overlapping variables of the results of the two algorithms. OSA, obstructive sleep apnea; BRI, body roundness index; BMI, body mass index.

The variables characterizing OSA severity worsening during follow-up were selected based on the combined results of LASSO regression, the Boruta algorithm, and random forest analysis in SHHS cohort (Figure 4). After comprehensive evaluation, the results identified key variables, including BRI, BMI, age, race/ethnicity, gender, neck circumference, snoring, tiredness, hypertension, and history of diabetes as the most important contributors.

The path diagram and coefficient trajectories of the LASSO regression illustrate the retained variables, indicating that BRI and BMI both exhibited strong contributions, with BRI demonstrating slightly greater coefficient stability (Figures 4A,B). The variable importance ranking derived from random forest analysis further supported these findings, showing that neck circumference, BRI, and BMI were the top-ranked features, with BRI consistently ranking higher than BMI (Figure 4C).

### Machine learning results

#### Model performance

We constructed three random forest models using the same covariate to assess the follow-up OSA severity over a 5-year follow-up in both the MrOS and SHHS cohorts using BRI, BMI and baseline features (Table 4). In the MrOS cohort, BRI achieved better model performance than BMI in fully adjusted Model, with an AUC of 0.67 (95% CI: 0.60–0.74) versus 0.57 (95% CI: 0.50–0.64, *p* = 0.04), and showed similar accuracy and F1 values. In Models 1 and 2, BRI also consistently demonstrated numerically higher AUCs than BMI (Model 1: 0.66 vs. 0.63; Model 2: 0.66 vs. 0.61).

**Table 4:** Weighted model performance of BRI and BMI for OSA severity worsening (delta degree > 0) during follow-up in two cohort.

Model		MrOS cohort		SHHS cohort	
		BRI	BMI	BRI	BMI
Model 1	AUC	0.66 (0.49, 0.76)	0.63 (0.50, 0.69)	0.71 (0.66, 0.76)*	0.66 (0.63, 0.74)*
	accuracy	0.61	0.58	0.62	0.59
	F1 value	0.54	0.46	0.55	0.49
Model 2	AUC	0.66 (0.48, 0.72)	0.61 (0.46, 0.70)	0.69 (0.64, 0.75)	0.67 (0.64, 0.74)
	accuracy	0.70	0.60	0.63	0.58
	F1 value	0.57	0.51	0.51	0.46
Model 3	AUC	0.67 (0.60, 0.74)*	0.57 (0.50, 0.64)*	0.73 (0.68, 0.78)*	0.68 (0.63, 0.74)*
	accuracy	0.60	0.55	0.63	0.60
	F1 value	0.50	0.44	0.58	0.46

Model 1 was unadjusted. Model 2 was adjusted for age, race, snore. Model 3 was adjusted for BRI, BMI, age, race, gender, neck circumference, snoring, tiredness, hypertension, and history of diabetes, inverse probability weighting was used to adjust for attrition due to either death or failure to attend follow-up visit. *p < 0.05 indicate levels of statistical significance.

**Figure 5:**
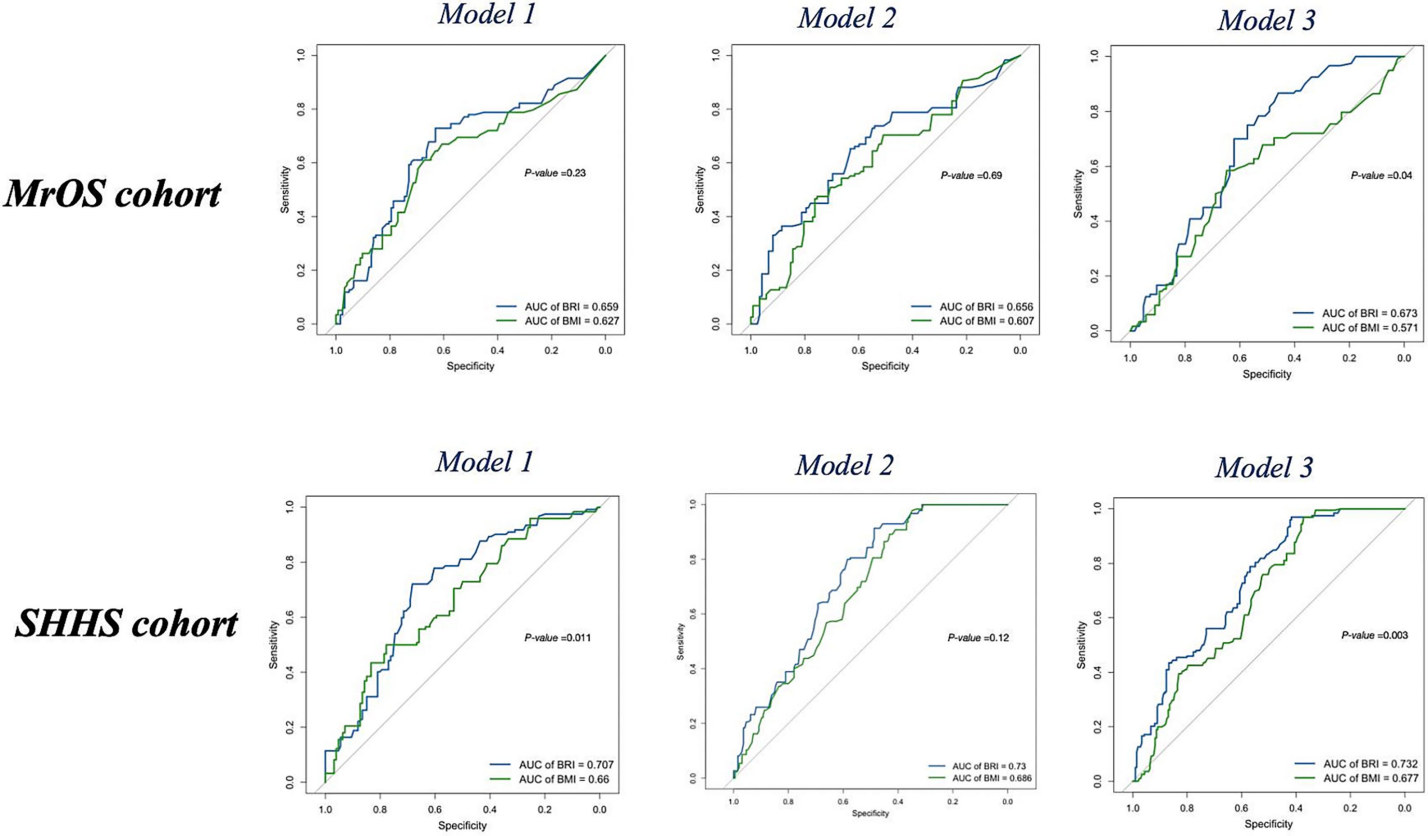
ROC curves of BRI (blue) and BMI (green) for follow-up OSA severity (delta degree > 0) in the MrOS cohort and SHHS cohort across Model 1, Model 2, and Model 3. BMI, body mass index; BRI, body roundness index; OSA, obstructive sleep apnea; MrOS, Osteoporotic Fractures in Men Study; SHHS, Sleep Heart Health Study; OR, odds ratio; AUC, area under the curve; ROC, receiver operating characteristic curve.

In the SHHS cohort, BRI showed higher AUCs than BMI in Model 1 (0.71 vs. 0.66; *p* = 0.011) and Model 3 (0.73 vs. 0.68; *p* < 0.01), along with higher accuracy and F1 values (Figure 5). Detailed performance metrics, including accuracy and F1 values for each model, are summarized in Table 4. NRI analyses showed that BRI provided better risk reclassification than BMI in the fully adjusted models of both cohorts (MrOS NRI_overall_ = 0.32, *p* < 0.05, SHHS NRI _overall_ = 0.20, *p* < 0.05) (Supplementary Table 12).

#### Model explanation

**Figure 6:**
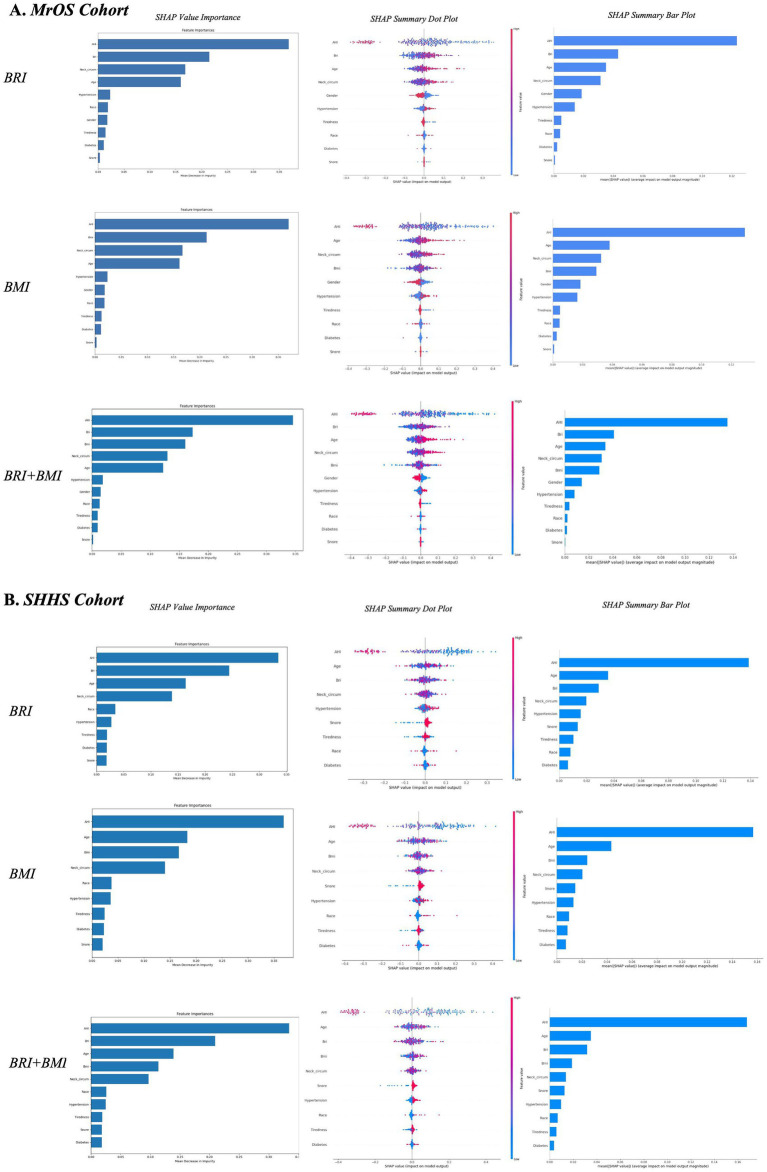
Global and local model explanations for BRI, BMI, and combined models in two cohort by the SHAP method **(A,B)**. SHAP value importance quantifies the contribution of each feature to the model’s estimated risk. SHAP summary bar plot: Displays the mean absolute SHAP values of each feature in descending order, reflecting their overall importance in the model. SHAP summary dot plot: Each dot represents a SHAP value for an individual observation. Dot color indicates feature value (red = high, blue = low), and vertical stacking shows density. Higher SHAP values correspond to greater contributions to the estimated risk of follow-up OSA severity worsening based on baseline features. BMI, body mass index; BRI, body roundness index; SHAP, SHapley Additive exPlanations; MrOS, Osteoporotic Fractures in Men Study; SHHS, Sleep Heart Health Study; OSA, obstructive sleep apnea.

To enhance the interpretability of the random forest models, we applied SHAP to both the MrOS and SHHS cohorts. Within each cohort, three model configurations were implemented: one including BRI, one including BMI, and one including both BRI and BMI simultaneously. As shown in Figure 5, global feature importance rankings consistently identified AHI, BRI, BMI, neck circumference, and age as the top features of follow-up OSA severity worsening across both cohorts (Figure 6). In models where BRI and BMI were evaluated separately, both indicators ranked prominently in terms of SHAP value importance across both cohorts, suggesting that BRI and BMI independently provide meaningful information to the model.

To enable direct comparison of their relative contributions, a joint model incorporating both BRI and BMI was further analyzed. In this setting, BRI exhibited consistently higher SHAP importance than BMI across both cohorts, indicating superior relevance. The SHAP summary plots further illustrated the direction and magnitude of feature effects on the model output, with red indicating higher feature values and blue indicating lower values. BRI showed a consistently strong and positive contribution to the model preformance, slightly higher than BMI, reinforcing its role as an important anthropometric indicator. Other features such as neck circumference and age also contributed moderately, whereas hypertension, race, tiredness, and snoring had minimal effects.

## Discussion

In this cross-sectional analysis, the body roundness index (BRI) consistently demonstrated more significant associations with the risk of baseline OSA than the BMI. In the follow-up models, BRI contributed more strongly than BMI to the classification of OSA severity during follow-up, indicating better discriminatory performance when baseline features were used. Furthermore, SHAP analyses indicated that BRI had more contribution than BMI to the models, enhancing its important role in classifying follow-up OSA severity.

Generally, body mass index is the most widely used measure for assessing obesity-related health risks. However, growing evidence suggests that visceral adiposity, rather than general obesity, plays a more critical role in the pathogenesis of OSA (24). Visceral adiposity independently increases the risk of OSA through increasing peripharyngeal soft tissue and exerts external mechanical loading on the upper airway, which is strongly linked to upper airway collapsibility and the pathophysiology of OSA, especially in severe OSA (25–27). In individuals with visceral fat obesity, the soft palate tends to shift backward and downward, while the tongue base moves closer to the posterior pharyngeal wall during inspiration. In the previous study, E Shinohara et al. utilized CT scan imaging to examine subcutaneous fat and visceral fat, further demonstrated that visceral adipose tissue area, and the ratio of visceral adipose tissue area to total adipose tissue area correlated with the severity of sleep apnea in obese subjects (28). Besides, the visceral fat accumulation elevates the diaphragm, leading to a reduction in lung volume, whereby pharyngeal narrowing would be induced, which causes the decrease in pharyngeal cross-sectional area. Since the effect of visceral fat on lung volume in a supine position will be evident, the upper airway may be liable to collapse during sleep. Another possibility is that the mass load of visceral fat may cause an increase in activities of the inspiratory muscles probably by restricting the motion of the diaphragm, resulting in the production of enough negative pressure to collapse the upper airway during the inspiratory phase.

Notably, aging promotes a shift of body fat from the subcutaneous to the visceral compartment independently of weight gain (29, 30), which is also associated with upper airway soft tissue enlargement and upper airway lengthening. This age-related shift is more pronounced in men than in women, resulting in a progressive accumulation of visceral fat in the abdominal region, which has been linked to increased cardiometabolic and respiratory risk, including a higher prevalence of OSA (31–33). Prior work has reported that anthropometric indices reflecting abdominal obesity were strongly associated with both OSA severity and cardiometabolic disease burden in patients with OSA (34). OSA, especially in moderate to severe forms, is associated with reduced aerobic capacity, impaired ventilatory and cardiovascular responses during exertion, while decreased exercise capacity might further exacerbate the metabolic burden and ventilatory instability which promotes OSA severity (35). This bidirectional relationship is further supported by interventional studies showing that CPAP therapy improves peak oxygen uptake and ventilatory efficiency in patients with severe OSA (36).

Visceral fat accumulation is closely related to glucose intolerance, hyperlipidemia, hypertension, and left ventricular dysfunction in obese subjects. As a superior indicator for evaluating visceral adiposity, BRI was found more valuable in identifying individuals at risk for severe OSA than BMI. Besides, BRI may more sensitively detect metabolically high-risk individuals including those with normal BMI but elevated visceral fat which are more likely to clinically significant OSA but may be underestimated by conventional screening criteria (37).

A statistically significant trend across BRI quartiles was observed, with increasing odds of OSA from Q2 to Q4. In contrast, for BMI, only the highest quartile (Q4) showed a significant association with baseline OSA risk in the fully adjusted model. Given that the MrOS cohort included only male participants, we conducted additional sex-stratified analyses in the SHHS cohort to evaluate the association between BRI and OSA risk in both sexes. When stratified by sex, BRI remained significantly associated with OSA in both men and women, but the magnitude of association was notably higher in males (MrOS: OR = 1.42, 95% CI: 1.26–1.61, *p* < 0.001, SHHS: OR = 1.42, 95% CI: 1.26–1.61, *p* < 0.001) compared to females (MrOS: OR = 1.15, 95% CI: 1.14–1.30; SHHS: OR = 1.15, 95% CI: 1.14–1.30, *p* < 0.001), suggesting that BRI may have a stronger relationship with OSA severity in men. These findings suggest a potential sex-specific difference in the physiological relevance of BRI in relation to OSA severity. This difference may be attributable to well-established sex-related variations in fat distribution. Men typically accumulate greater amounts of visceral and upper-body fat, whereas women are more prone to peripheral and subcutaneous fat deposition. Hunter et al. showed that visceral fat doubles in men from the 3rd to the 7th decade (38). Moreover, sex hormones and anatomical differences may modulate the impact of adiposity on OSA risk in women. Estrogen and progesterone have been reported to confer protective effects on upper airway stability, especially in premenopausal women, potentially attenuating the influence of central fat accumulation on OSA development (39, 40). Our findings support the need for sex-specific interpretation of adiposity indices in OSA risk assessment.

In our analysis, we further examined the clinical relevance of BRI and BMI by incorporating it, along with other clinical variables, including age, race/ethnicity, gender, neck circumference, snoring, tiredness, hypertension, and history of diabetes, into machine learning models. These variables are largely consistent with those included in the STOP-Bang framework, a widely used and validated screening tool for assessing OSA risk. The original STOP-Bang questionnaire comprises eight binary items: snoring, tiredness, observed apnea, high blood pressure, BMI, age, neck circumference, and gender, reflecting clinically accessible indicators of OSA risk (41). In the present analysis, diabetes was additionally incorporated, given its well-established association with OSA and its role in cardiometabolic risk stratification. To preserve clinical interpretability and facilitate potential translational applications, we employed random forest models based on these covariates to assess OSA severity worsening using baseline features in two large, community-based cohorts. Random forests offer a balance between model performance and explain ability, particularly through variable importance metrics, making them suitable for translational applications in risk stratification and clinical decision support (42, 43). Individuals with higher BRI at baseline were more likely to experience increases in AHI and more severe OSA categories during follow-up. These findings highlight the meaningful contribution of visceral adiposity, as estimated by BRI, to the long-term changes of OSA severity. SHAP analysis further supported these findings, showing that BRI consistently contributed more to model than BMI across both cohorts. Higher BRI values had a stronger and more stable positive impact on severe OSA risk, while the contribution of BMI diminished when BRI was included, suggesting its limited incremental value. The consistent association between baseline BRI and future OSA worsening suggests its utility in early clinical risk assessment and the design of individualized prevention strategies. Although our model shares several variables with the STOP-Bang questionnaire, it additionally includes diabetes. Given that BMI is a component of STOP-Bang, replacing it with a more physiologically relevant index such as BRI, may improve the accuracy of OSA risk stratification. Further studies are needed to assess the value of integrating alternative anthropometric measures into existing screening frameworks.

Our study has several limitations. First, although we adjusted for multiple covariates, residual confounding from unmeasured lifestyle or metabolic factors cannot be excluded. Second, the MrOS cohort consists of older men, which may limit the generalizability of sex-specific findings despite validation in the mixed-gender SHHS cohort. Third, feature selection was based mainly on SHHS, but similar feature patterns observed in cohort-specific analyses support the stability of these features. These analyses were intended to compare the performance of BRI and BMI for OSA severity worsening based on baseline characteristics, which is not causal or prognostic modelling. Despite the use of inverse probability weighting to address differential loss to follow-up, the possibility of residual bias related to attrition cannot be fully excluded. Future studies should evaluate the stability of feature selection and model performance across diverse populations.

## Conclusion

In sum, across these two community-based cohorts, BRI was more associated with severe baseline OSA than BMI and provided closer association of future worsening OSA severity. Incorporating BRI into machine learning models based on baseline features enhanced their ability to identify individuals at higher risk of subsequent OSA severity increases. Further prospective studies with external validation are needed to confirm these findings and to explore the integration of BRI into precision-based screening strategies.

## Data Availability

The original contributions presented in the study are included in the article/Supplementary material, further inquiries can be directed to the corresponding author.
