# Supplementary material for: Associations of body roundness index and body mass index with obstructive sleep apnea: evidence from two cohorts study
Source: Front Neurol. 2025 Dec 16;16:1709205. doi: 10.3389/fneur.2025.1709205 (PMC12750020; doi:10.3389/fneur.2025.1709205)
Supplement: Supplementary file 1 [file Supplementary_file_1.docx]

**Supplement materials**


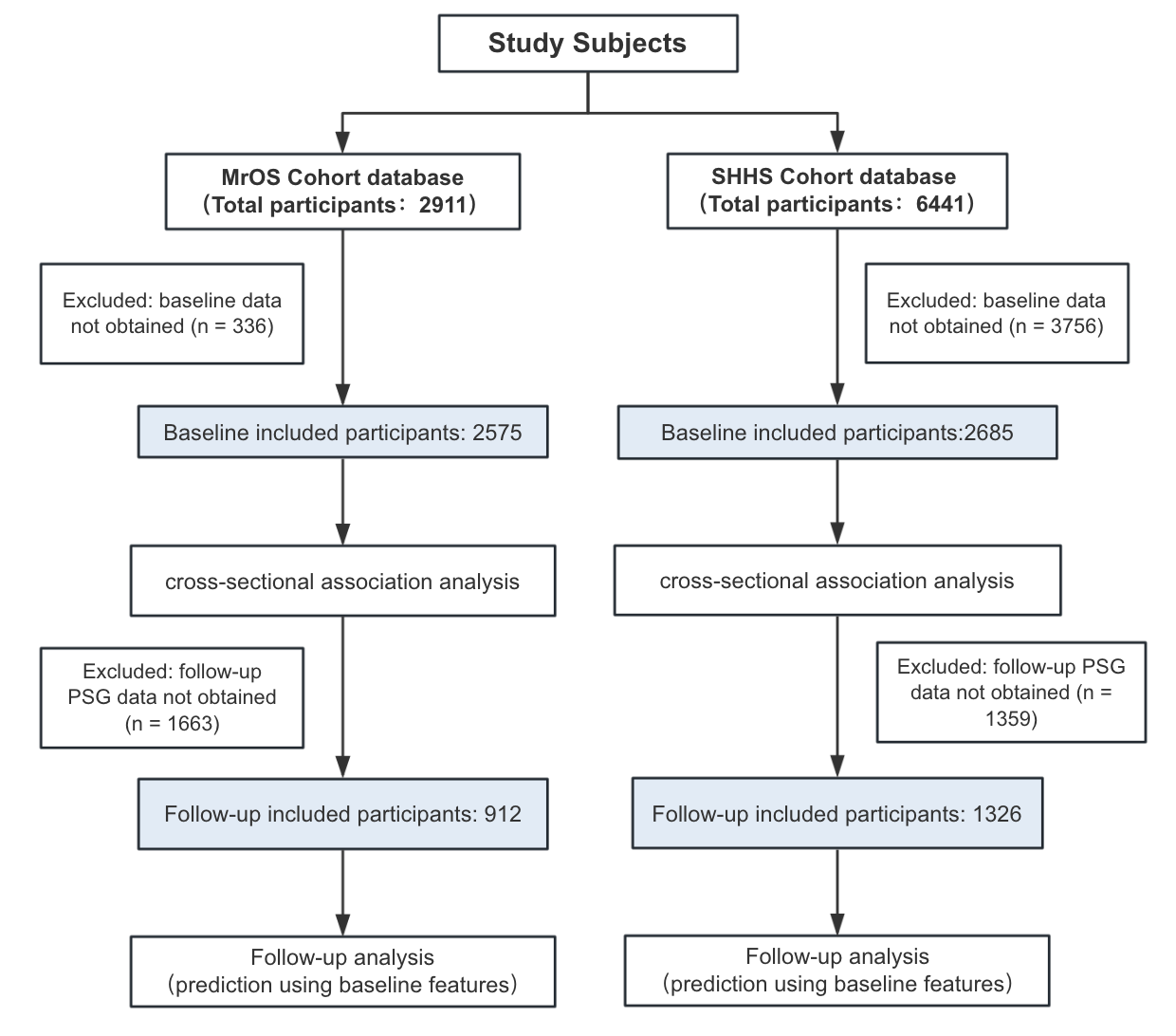


**sFigure 1. Flowchart of the study, Osteoporotic Fractures in Men Study, MrOS, Sleep Heart Health Study, SHHS.**

**sTable1 Stratified analyses of cohort and adiposity indicators (BRI and BMI) in baseline and follow-up models**

| cohort × adiposity indicator | Estimate | Std. Error | z value | P | LR Chisq |
| --- | --- | --- | --- | --- | --- |
| Cross-sectional models |  |  |  |  |  |
| cohort × BRI | -0.096 | 0.057 | -1.57 | 0.10 | 2.78 |
| cohort × BMI | -0.010 | 0.020 | -0.41 | 0.63 | 0.23 |
| Follow-up models |  |  |  |  |  |
| cohort × BRI | 0.040 | 0.092 | 0.44 | 0.66 | 0.19 |
| cohort × BMI | -0.0048 | 0.034 | -0.14 | 0.89 | 0.021 |

Stratified analyses assessing whether the associations and predictive performance of Body Roundness Index (BRI) and Body Mass Index (BMI) differed across cohorts (MrOS and SHHS). No significant interactions were observed (P > 0.05), indicating no evidence of heterogeneity between cohorts.

**Random Forest Model parameters**

All Random Forest models were implemented using the *randomForest* package in R (version 4.3.0). The hyperparameters were provided to ensure reproducibility and transparentcy.

**sTable2 Random Forest Model Parameters**

| Parameter | Description | Value |
| --- | --- | --- |
| n_estimators | Number of trees | **500** |
| mtry | Variables sampled per split | **2** |
| max_depth | Maximum depth | **Unlimited** |
| criterion | Splitting criterion | **Gini impurity** |
| class_weight approach | How class weights were handled | **Sampling-based outcome weighting** |
| variable importance | Importance metric | **Gini importance** |

**Class weight**

Class imbalance was addressed through sampling-based outcome weights. These weights were then multiplied by the inverse probability weights (IPW) to create the final analytic weight.

Final analytic weight = Class weight × IPW

This ensures the model accounts for both attrition and outcome imbalance.

**Inverse probability weight**

Inverse probability weighting (IPW) [1, 2]was used to account for the probability that each participant contributed follow-up PSG data. The weighting strategy followed a two-step framework commonly used in population-based sleep and aging cohorts, adapted for the structure of MrOS and SHHS cohort. Logistic regression models were used to estimate each participant’s probability of (1) being alive at the time of follow-up PSG and (2) completing the follow-up PSG, based on baseline sleep measures and covariates. Stabilized weights were winsorized at the 1st and 99th percentiles to reduce the influence of extreme values.

For subsequent analyses, analytic weights were generated by multiplying the stabilized IPW with the sampling-based outcome weights used to maintain class balance in the training data. These cohort-specific weight distributions are summarized in Supplementary Table S3. The final analytic weights were incorporated into all weighted regression models and machine-learning procedures.

**sTable3 Inverse probability weight Parameters**

| Cohort | Weight Component | Median [IQR] |
| --- | --- | --- |
| **MrOS** | Class weight | **0.731 [0.850]** |
|  | Inverse probability weight (IPW) | **2.532 [1.112]** |
|  | Final analytic weight (IPW × class weight) | **2.075 [1.527]** |
| **SHHS** | Class weight | **0.683 [1.186]** |
|  | Inverse probability weight (IPW) | **1.964 [0.484]** |
|  | Final analytic weight (IPW × class weight) | **1.169 [1.912]** |

**sTable4 Unweighted model performance of BRI and BMI for OSA severity worsening (delta degree > 0) during follow-up in two cohort.**

|  | | MrOS cohort | | SHHS cohort | |
| --- | --- | --- | --- | --- | --- |
| delta OSA degree >0). | | BRI | BMI | BRI | BMI |
| Model 1 | AUC | 0.73 (0.65,0.80) | 0.61 (0.52,0.70) | 0.70 (0.63,0.76) | 0.65(0.50,0.70) |
|  | accuracy | 0.60 | 0.58 | 0.59 | 0.55 |
|  | F1 value | 0.50 | 0.46 | 0.43 | 0.40 |
| Model 2 | AUC | 0.72(0.65,0.79) | 0.62(0.53,0.72) | 0.71(0.64,0.75) | 0.67(0.62,0.73) |
|  | accuracy | 0.69 | 0.56 | 0.60 | 0.54 |
|  | F1 value | 0.45 | 0.40 | 0.44 | 0.42 |
| Model 3 | AUC | 0.74(0.62, 0.77) * | 0.61(0.52,0.70) * | 0.74 (0.66, 0.76)* | 0.69(0.60, 0.71) * |
|  | accuracy | 0.59 | 0.55 | 0.58 | 0.57 |
|  | F1 value | 0.43 | 0.40 | 0.39 | 0.45 |

Model 1 was unadjusted. Model 2 was adjusted for age, race, snore, gender. Model 3 was adjusted for BRI, BMI, age, race, gender, neck circumference, snoring, tiredness, hypertension, and history of diabetes

**Variance inflation factors (VIFs)**

**sTable5** VIF values for models including BRI in SHHS cohort

| Variables | Vif |
| --- | --- |
| BRI | 1.58 |
| Age | 1.08 |
| **gender** | 2.31 |
| neck circumference | 2.29 |
| Race | 1.03 |
| snoring | 1.02 |
| tiredness | 1.02 |
| hypertension, | 1.10 |
| history of diabetes | 1.07 |

**sTable6** VIF values for models including BRI in SHHS cohort

| Variables | Vif |
| --- | --- |
| BMI | 2.03 |
| Age | 1.52 |
| **gender** | 2.42 |
| neck circumference | 2.84 |
| Race | 1.03 |
| snoring | 1.02 |
| tiredness | 1.03 |
| hypertension, | 1.12 |
| history of diabetes | 1.08 |

**sTable7** VIF values for models including BRI and BMI in SHHS cohort

| Variables | Vif |
| --- | --- |
| BRI | 3.37 |
| BMI | 4.42 |
| Age | 1.25 |
| **gender** | 2.47 |
| neck circumference | 2.87 |
| Race | 1.03 |
| snoring | 1.03 |
| tiredness | 1.02 |
| hypertension, | 1.12 |
| history of diabetes | 1.08 |

**sTable8** VIF values for models including BRI in MrOS cohort

| Variables | Vif values |
| --- | --- |
| BRI | 1.28 |
| Age | 1.06 |
| neck circumference | 1.01 |
| Race | 1.01 |
| snoring | 1.03 |
| tiredness | 1.01 |
| hypertension, | 1.03 |
| history of diabetes | 1.03 |

**sTable9** VIF values for models including BMI in MrOS cohort

| Variables | Vif values |
| --- | --- |
| BMI | 1.41 |
| Age | 1.06 |
| neck circumference | 1.02 |
| Race | 1.01 |
| snoring | 1.03 |
| tiredness | 1.01 |
| hypertension, | 1.02 |
| history of diabetes | 1.03 |

**sTable10** VIF values for models including BRI and BMI in MrOS cohort

| Variables | Vif values |
| --- | --- |
| BRI | 2.11 |
| BMI | 2.31 |
| Age | 1.08 |
| neck circumference | 1.02 |
| Race | 1.02 |
| snoring | 1.03 |
| tiredness | 1.01 |
| hypertension, | 1.03 |
| history of diabetes | 1.03 |

**Sensitivity analysis**

AHI was examined for distributional normality, normally distributed variables were analyzed as continuous values, whereas right-skewed variables (including AHI) were log-transformed to reduce skewness and approximate normality. In sensitivity analyses, we used log-transformed AHI as a continuous variable to assess robustness to outcome specification. BRI remained significantly associated with higher AHI, and the estimates aligned closely with those obtained using categorical AHI definitions.

**sTable11 Associations of BRI and BMI with AHI in MrOS cohort and SHHS cohort**

|  | β (Estimate) | Std. Error | p value |
| --- | --- | --- | --- |
| **MORS** |  |  |  |
| **BRI** | 0.052(0.036, 0.068) | 0.0083 | <0.0001 |
| **BMI** | 0.025(0.019,0.031) | 0.0031 | <0.0001 |
| **SHHS** |  |  |  |
| **BRI** | 0.082(0.064, 0.10) | 0.0094 | <0.0001 |
| **BMI** | 0.039(0.032, 0.046) | 0.0036 | <0.0001 |

Regression coefficients (β), standard errors, and *p*-values from multivariable models treating log-transformed AHI as a continuous outcome in the MrOS and SHHS cohorts.

**NRI Predictive performance.**

The net reclassification improvement (NRI) is an increasingly used measure for evaluating improvements in risk prediction and was applied here following the framework introduced by previous studies [3-5]. In our study, NRI was used to quantify whether BRI based model performed better compared with a BMI based model in assigning individuals to higher predicted risk when the OSA severity worsened, and to lower predicted risk when the OSA severity remain stable. We applied the continuous category-free NRI, which examines whether predicted risk moves in the correct direction for each individual. To quantify uncertainty in the NRI estimates, we followed recommended practice and used bootstrap resampling. The distribution of these resampled values was then used to derive 95% confidence intervals and two-sided p-values, allowing us to assess whether the observed NRI differed meaningfully from zero. This approach provides a robust estimate of the comparative value of BRI versus BMI in predicting which individuals are more likely to have worsening OSA severity.

**sTable12 NRI Predictive performance**

| Cohort | Model | NRI_event | NRI_nonevent | NRI_overall | NRI_overall_ 95% CI | *p*-value |
| --- | --- | --- | --- | --- | --- | --- |
| MrOS | Model 1 | 0.033 | -0.17 | **-0.14** | **(-0.38,0.097)** | **0.53** |
|  | Model 2 | 0.23 | -0.072 | 0.160 | (-0.095,0.396) | 0.48 |
|  | Model 3 | 0.28 | 0.040 | 0.32 | (0.080,0.47） | 0.016 |
| SHHS | Model 1 | 0.16 | 0.097 | 0.25 | (0.088,0.44) | 0.008 |
|  | Model 2 | 0.092 | 0.12 | 0.21 | (0.015,0.42) | 0.029 |
|  | Model 3 | 0.14 | 0.067 | 0.20 | (0.010,0.40) | 0.036 |

NRI, net reclassification index


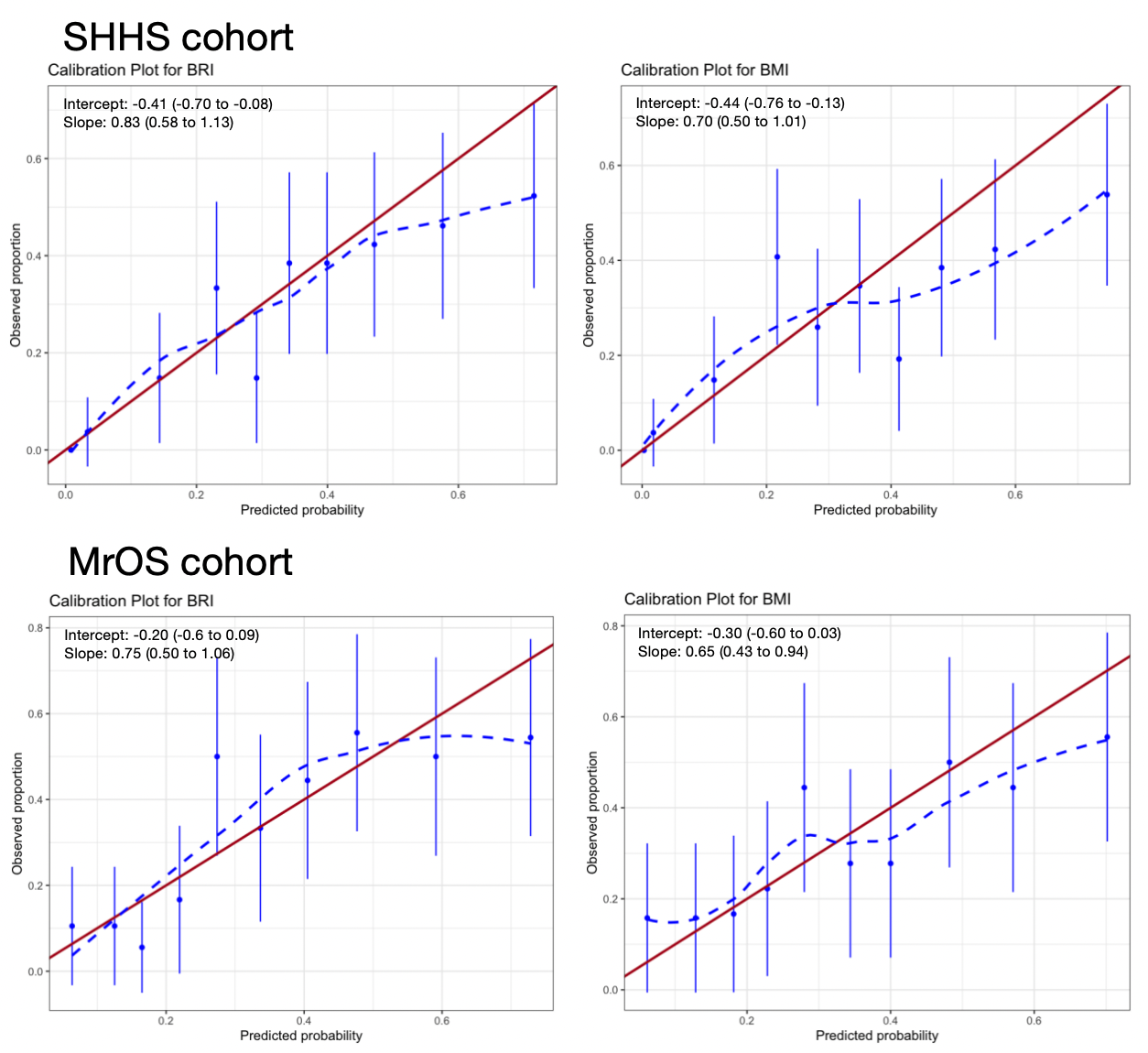


sFigure 2. Calibration plots of Random Forest models assessing OSA severity worsening using BRI and BMI in the SHHS and MrOS cohorts

Calibration performance of Random Forest models for assessing worsening of OSA severity at follow-up based on baseline features. The red diagonal line represents perfect calibration. The blue dashed line indicates the observed proportion of worsening OSA within each predicted-risk decile, with 95% confidence intervals. Intercept and slope values (with 95% CI) are shown within each panel.

Reference

1. Seaman SR, White IR: **Review of inverse probability weighting for dealing with missing data**. *Stat Methods Med Res* 2011, **22**(3):278-295.

2. Hernán MA, Hernández-Díaz S, Robins JM: **A structural approach to selection bias**. *Epidemiology* 2004, **15**(5):615-625.

3. Leening MJG, Vedder MM, Witteman JCM, Pencina MJ, Steyerberg EW: **Net reclassification improvement: computation, interpretation, and controversies: a literature review and clinician's guide**. *Ann Intern Med* 2014, **160**(2):122-131.

4. Jewell ES, Maile MD, Engoren M, Elliott M: **Net Reclassification Improvement**. *Anesth Analg* 2016, **122**(3):818-824.

5. Pencina MJ, D'Agostino RB, D'Agostino RB, Vasan RS: **Evaluating the added predictive ability of a new marker: from area under the ROC curve to reclassification and beyond**. *Stat Med* 2008, **27**(2).
